# Comparison of Bacterial Burden and Cytokine Gene Expression in Golden Hamsters in Early Phase of Infection with Two Different Strains of *Leptospira interrogans*

**DOI:** 10.1371/journal.pone.0132694

**Published:** 2015-07-06

**Authors:** Rie Fujita, Nobuo Koizumi, Hiromu Sugiyama, Rina Tomizawa, Ryoichi Sato, Makoto Ohnishi

**Affiliations:** 1 Graduate School of Bio-Applications & Systems Engineering, Tokyo University of Agriculture and Technology, Koganei, Tokyo, Japan; 2 Department of Bacteriology I, National Institute of Infectious Diseases, Shinjuku, Tokyo, Japan; 3 Department of Parasitology, National Institute of Infectious Diseases, Shinjuku, Tokyo, Japan; Cornell University, UNITED STATES

## Abstract

Leptospirosis, a zoonotic infection with worldwide prevalence, is caused by pathogenic spirochaetes of *Leptospira* spp., and exhibits an extremely broad clinical spectrum in human patients. Although previous studies indicated that specific serovars or genotypes of *Leptospira* spp. were associated with severe leptospirosis or its outbreak, the mechanism underlying the difference in virulence of the various *Leptospira* serotypes or genotypes remains unclear. The present study addresses this question by measuring and comparing bacterial burden and cytokine gene expression in hamsters infected with strains of two *L*. *interrogans* serovars Manilae (highly virulent) and Hebdomadis (less virulent). The histopathology of kidney, liver, and lung tissues was also investigated in infected hamsters. A significantly higher bacterial burden was observed in liver tissues of hamsters infected with serovar Manilae than those infected with serovar Hebdomadis (p < 0.01). The average copy number of the leptospiral genome was 1,302 and 20,559 in blood and liver, respectively, of hamsters infected with serovar Manilae and 1,340 and 4,896, respectively, in hamsters infected with serovar Hebdomadis. The expression levels of *mip1alpha* in blood; *tgfbeta*, *il1beta*, *mip1alpha*, *il10*, *tnfalpha* and *cox2* in liver; and *tgfbeta*, *il6*, *tnfalpha* and *cox2* in lung tissue were significantly higher in hamsters infected with serovar Manilae than those infected with serovar Hebdomadis (p < 0.05). In addition, infection with serovar Manilae resulted in a significantly larger number of hamsters with *tnfalpha* upregulation (p = 0.04). Severe distortion of tubular cell arrangement and disruption of renal tubules in kidney tissues and hemorrhage in lung tissues were observed in Manilae-infected hamsters. These results demonstrate that serovar Manilae multiplied more efficiently in liver tissues and induced significantly higher expression of genes encoding pro- and anti-inflammatory cytokines than serovar Hebdomadis even in tissues for which a significant difference in leptospiral load was not observed. In addition, our results suggest a serovar Manilae-specific mechanism responsible for inducing severe damage in kidneys and hemorrhage in lung.

## Introduction

Leptospirosis is a zoonotic infection caused by pathogenic spirochaetes of *Leptospira* spp., and has worldwide prevalence. The disease constitutes an important public health problem in tropical regions, particularly in South and Southeast Asia as well as Latin America [1, 2]. Leptospires colonize the proximal renal tubules and are excreted in the urine of maintenance hosts [2, 3]. Human leptospirosis is chiefly transmitted by exposure to water or soil contaminated with the urine of infected animals [3].

Human leptospirosis is an acute febrile illness with an extremely broad clinical spectrum ranging from mild influenza-like illness to severe disease forms characterized by jaundice, bleeding, renal failure, and death [2, 4]. In recent years, a severe form of the disease characterized by pulmonary hemorrhage, known as leptospirosis-associated severe pulmonary hemorrhage syndrome, has gained attention as an important cause of mortality [2, 5]. However, a majority of infected patients develop subclinical or very mild illness [6, 7], with only 5%–10% of the patients potentially developing a severe form of the disease [2, 8]. The infecting inoculum dose of leptospires, pre-existing immunity because of a previous infection and/or genetic determinants in patients pertaining to innate or acquired immunity are considered possible determinants of disease severity [9].

Several studies have demonstrated the association between serum levels of cytokines and clinical outcome in patients with leptospirosis [10–15]. In contrast to mild leptospirosis, a broad activation of both pro- and anti-inflammatory cytokines was observed to occur in patients with severe disease, which is similar to the cytokine storm observed during sepsis caused by other bacteria [10]. Of the several cytokines with elevated levels, IL-6 and IL-10 are independent predictors of death [10]. Leptospirosis cases with fatal outcomes exhibited higher IL-10 but lower TNF-α levels than survivors, and a positive correlation was identified between the IL-10/TNF-α ratio and fatal outcomes [11]. However, another group reported high levels of TNF-α in severe disease as well as association of high IL-10/TNF-α ratio with lower disease severity [12, 13]. Other cytokines, such as soluble ST2 and long pentraxin PTX3, have been shown to be associated with severe leptospirosis [14, 15]. In an animal model of leptospirosis, genes for both pro- and anti-inflammatory cytokines, including *tnfalpha*, *il1alpha*, *cox2* and *il10*, were significantly upregulated in the blood of dead compared with recovered hamsters [16].

In addition to host immune responses, virulence determinants of the infecting *Leptospira* strains also affect disease severity. Severe leptospirosis is frequently caused by strains of the serogroup Icterohaemorrhagiae, although the association of distinct clinical syndromes with specific serogroups has been refuted [8, 17]. In addition to the serological phenotypes, a specific clone belonging to *L*. *interrogans* serovars Copenhageni and Autumnalis has been shown to be associated with severe pulmonary hemorrhage syndrome in Brazil and outbreaks of leptospirosis in Thailand, respectively [18, 19]. However, the mechanism underlying the differences in virulence of various *Leptospira* serotypes or genotypes remains unclear.

Our previous study showed the difference in virulence of two *L*. *interrogans* strains belonging to different serovars in hamsters [20]; a strain of *L*. *interrogans* serovar Manilae caused lethality in hamsters, while 10^7^ cells of a strain of *L*. *interrogans* serovar Hebdomadis failed to cause death [20]. In the present study, we aimed to gain insights into the mechanism underlying the difference in virulence of the strains belonging to different *L*. *interrogans* serovars. With this goal, we compared the expression of cytokine genes and leptospiral burden in tissues of hamsters infected with strains of *L*. *interrogans* serovars Manilae or Hebdomadis.

## Materials and Methods

### Strains of *L*. *interrogans* and culture conditions

*L*. *interrogans* serovar Manilae strain UP-MMC-NIID and *L*. *interrogans* serovar Hebdomadis strain OP84 [20, 21] were employed in the present study. The strains were cultured in liquid modified Korthof’s medium supplemented with 10% rabbit serum at 30°C [3]. The strains were stored in korthof’s medium containing 10% glycerol at −80°C and subjected to *in vivo* passage at least once every two years during which the serovar Manilae strain maintained lethality. The virulence of the strains in hamsters was examined prior to each infection experiment, and leptospires were recovered from kidney tissues of infected hamsters. Each strain was passaged *in vitro* fewer than three times for use in infection experiments. All animal experiments were approved by the Animal Research Committee of the National Institute of Infectious Diseases (Tokyo, Japan).

### Experimental infection of hamsters

Six-week-old female specific-pathogen-free (SPF) golden hamsters (*Mesocricetus auratus*) were employed in this study. Hamsters were purchased from SLC (Shizuoka, Japan) and three or four animals were housed in one cage according to the Institute for Laboratory Animal Research standard. All animals had unfettered access to irradiated standard diet and sterile drinking water and were maintained in a 14 h-light/10 h-dark cycle at the SPF ward. No apparent abnormalities were observed in all animals prior to infection experiments. The number of leptospiral cells was enumerated using a counting chamber of 0.010-mm depth (Nitirin, Tokyo, Japan). Hamsters were subjected to intraperitoneal inoculation with 1 × 10^6^ cells of the *L*. *interrogans* strains (of serovars Manilae or Hebdomadis) in the log phase suspended in 500 μl of Korthof’s medium. Hamsters inoculated with the same volume of medium alone were employed as controls. For preliminary time-course experiments, three hamsters inoculated with each *Leptospira* strain were euthanized by isoflurane inhalation at 12, 24, 48, 72, and 96 h postinoculation (pi). For the main experiments, seven and eight hamsters inoculated with the strains of serovars Manilae and Hebdomadis, respectively, were similarly euthanized at 96 h pi. Inoculation of one hamster with the strain belonging to serovar Manilae was not successful and was excluded from this study. All animals survived with no complications in each experiment. Immediately following euthanasia, whole blood was collected by cardiac puncture; 50 μl of blood was inoculated into 4 ml of Korthof’s medium and was cultured as described above. In addition, 100 μl of blood was collected in microtubes containing 100 μl of PBS and 20 μl of proteinase K (DNeasy Blood & Tissue Kit, Qiagen, Hilden, Germany) and was immediately subjected to DNA extraction (detailed below). For RNA extraction, 500 μl of blood was collected in RNAprotect Animal Blood Tubes (Qiagen) and stabilized at room temperature for 2 h prior to storage at −20°C. Following the collection of blood, the hamsters were dissected and kidney, liver, and lung tissues were collected for DNA/RNA extraction and histological examination, as subsequently detailed. Three-fourths of the kidney tissue, ventral medial lobe of the liver tissue, and left lobe of the lung tissue were stored at −20°C in 2.5–3.5 ml of RNA*later* (Ambion, Austin, TX) until DNA or RNA extraction. The remainder of each tissue was fixed with 4% paraformaldehyde, dehydrated, and embedded in paraffin followed by sectioning and staining with hematoxylin and eosin.

### DNA extraction

DNA was extracted from 100 μl of whole blood and approximately 25 mg samples of renal cortex, middle part of the ventral medial lobe of the liver, and the dorsal part of left lobe of the lung tissues using DNeasy Blood & Tissue Kit (Qiagen). The concentration of the extracted DNA samples was determined using NanoDrop Lite Spectrophotometer (Thermo-Scientific, Rockford, IL), and the DNA samples were diluted to a concentration of 100 ng/μl with Tris-EDTA (TE) buffer (pH 8.0).

### Total RNA extraction and cDNA synthesis

Total RNA was extracted from 500 μl of whole blood and approximately 20 mg samples of renal cortex, middle part of the ventral medial lobe of the liver, and the dorsal part of left lobe of the lung tissues using RNeasy Protect Animal Blood Kit (Qiagen) and RNeasy Plus Mini Kit (Qiagen), respectively. The concentration of the extracted RNA samples was determined using NanoDrop Lite Spectrophotometer, and 500–1500 ng of RNA was employed for cDNA synthesis using ReverTra Ace qPCR RT Master Mix with gDNA remover (Toyobo Co. Ltd., Tokyo, Japan). cDNA concentration was adjusted to 100 ng/μl with RNase-free water for relative quantification (2^−ΔΔCt^ method) or with TE buffer for absolute quantification.

### Real-time PCR analysis

Real-time PCR amplification and analysis was conducted using LightCycler Nano instrument and software version 1.1 (Roche Life Sciences, Rotkreuz, Switzerland). Reactions were conducted in 20-μl volumes containing THUNDERBIRD SYBR qPCR Mix (Toyobo Co. Ltd.). Primer sequences and concentrations are listed in Table 1. The PCR conditions included initial denaturation at 95°C for 60 s, followed by 40 cycles of amplification of target sequences at 95°C for 10 s, and 60°C for 30 s. Following the amplification program, melting curve analysis was conducted to assess the specificity of the amplified products.

**Table 1:** Sequences of primers employed for real-time PCR.

Gene	Primer Sequence (5′→3′)		Ref. (Accession No.)	Primer conc. (nM)		Method^a^	Amplification efficiency (%)	Amplification efficiency *of rpl18* (%)^b^
	Forward	Reverse		Forward	Reverse			
RPL18	GTTTATGAGTCGCACTAACCG	TGTTCTCTCGGCCAGGAA	[36]	300	300	―	―	―
IP10/CXCL10	CTCTACTAAGAGCTGGTCC	CTAACACACTTTAAGGTGGG	[37]	200	200	C	90.42	93.76
TGFβ	TGTGTGCGGCAGCTGTACA	TGGGCTCGTGAATCCACTTC	[36]	600	600	C	92.79	93.76
IL-1β	GGCTGATGCTCCCATTCG	CACGAGGCATTTCTGTTGTTCA	[36]	200	200	C	94.27	93.76
MIP1α/CCL3	CTCCTGCTGCTTCTTCTA	TGGGTTCCTCACTGACTC	[37]	200	200	C	97.11	93.66
IL-6	CCTGAAAGCACTTGAAGAATTCC	GGTATGCTAAGGCACAGCACACT	[36]	600	600	C	97.37	97.79
IL-10	GTTGCCAAACCTTATCAGAAATGA	TTCTGGCCCGTGGTTCTCT	[36]	600	600	C	93.87	95.79
iNOS	TGGCAGGATGGGAAACTGA	GCACCGCTTTCACCAAGACT	[36]	300	300	C	91.19	93.95
TNF-α	GGAGTGGCTGAGCCATCGT	AGCTGGTTGTCTTTGAGAGACATG	[36]	300	300	C	90.94	94.35
COX-2	CTTCCTCCTGTGGCTGATGACT	TCTTTCGAATCAGGAAGCTCCTT	This study (AF345331) ^c^	600	600	C	90.77	91.61
GAPDH	CCGAGTATGTTGTGGAGTCTA	GCTGACAATCTTGAGGGA	[37]	900	900	―	―	―
IFN-γ	GGCCATCCAGAGGAGCATAG	TTTCTCCATGCTGCTGTTGAA	[36]	600	600	A	―	―
IL-2	GTGCACCCACTTCAAGCTCTAA	AAGCTCCTGTAAGTCCAGCAGTAAC	[36]	900	900	A	―	―
IL-4	CCAGGTGCTTCGCAAGTTTT	TCTTTGAGAACCCTGGAATTGTTC	This study (XM_005067769)^c^	900	900	A	―	―
*flaB*	CTTACGARAGATCATGAAGCAGAG	TGTTTTGTGGTCAGCGAGACA	This study	900	900	A	―	―

^a^ C: Comparative cycle threshold (Ct) method (2^−ΔΔCt^ method); A: Absolute quantification

^b^ Efficiency of RPL18 amplification simultaneously with that of each target gene

^c^ Accession number

### Quantification of leptospiral DNA in animal tissues

Absolute quantification of the gene *flaB* encoding leptospiral flagellin was employed for quantifying leptospiral DNA in hamster tissues under the abovementioned conditions. A standard curve for quantification was generated using serially diluted genomic DNA of known concentration extracted from *L*. *interrogans* serovar Manilae strain. The housekeeping gene glyceraldehyde 3-phosphate dehydrogenase (*gapdh*) from a hamster was simultaneously quantified for verifying the quality and quantity of the extracted DNA. Results were expressed as genome equivalents per microliter of whole blood or per 200 ng DNA from other tissues, and the number of genome equivalents in the reaction mixture was calculated considering a genome size of 4.6 Mbp (corresponding to *L*. *interrogans* Fiocruz L1-130 [22]). Experiments were performed in duplicate using two independently extracted DNA samples for each tissue of infected hamsters. Statistical comparison of the leptospiral genome copy numbers observed in tissues infected with serovars Manilae and Hebdomadis was conducted using Mann–Whitney U test.

### Cytokine gene expression analysis

The gene encoding ribosomal protein L18 (RPL18) was selected as an internal reference because its expression was more stable in all the tissues of infected and control hamsters compared with GAPDH or β-2-microglobulin genes (data not shown). The amplification efficiency (AE) for each primer set was determined by generating a standard curve from serial dilutions of cDNA synthesized from RNA extracted from *Leptospira*-inoculated hamster tissues. AE was calculated using the formula AE = 10^[−1/slope]^ − 1 (the slope was determined from the correlation between the Ct value and template concentration). The differences between the AE of each target gene (including nine genes, with the exception of *il2*, *il4*, and *ifngamma*) and the AE of *rpl18* (Table 1) did not exceed 5%. Thus, quantification of the expression of these nine target genes was conducted using the comparative cycle threshold (Ct) method (2^−ΔΔCt^ method) using *rpl18* as a calibrator gene. For these nine target genes, relative gene expression was calculated as the ratio of the level of expression in infected to control hamsters. For certain genes whose expression was not detectable in control hamsters, the gene expression level in infected hamsters was calculated as the ratio of the level of expression in hamsters infected with serovar Manilae to those infected with serovar Hebdomadis. The expression of *il2*, *il4*, and *ifngamma* was determined by absolute quantification using a standard curve generated by amplifying serial dilutions of known concentrations of plasmids containing each target sequence using the primer sets listed in Table 1. The cut-off value for real-time PCR was set as Ct35. Experiments were performed in duplicate using two independently extracted RNA samples for each tissue of infected hamsters. Statistical comparison of cytokine gene expression in tissues infected with serovars Manilae and Hebdomadis was conducted using Mann–Whitney U test.

## Results

### Temporal changes in bacterial burden and cytokine gene expression in hamsters infected with strains of two different *L*. *interrogans* serovars

A previous study from our group demonstrated that hamsters infected with *L*. *interrogans* serovar Manilae (inoculum dose of 1 × 10^6^ cells) died at 100–120 h pi. Therefore, as a preliminary experiment, leptospiral burden and the expression of cytokine genes were measured in tissues of infected hamsters at 12, 24, 48, 72, and 96 h pi.

Leptospiral burden in tissues was determined as genome equivalents by real-time PCR targeting the leptospiral *flaB* gene. *flaB* was detected in blood and the liver tissue of hamsters infected with either of the two strains from 48 h pi onwards but only at 96 h pi in kidney and lung tissues (S1 Fig).

The expression of 12 cytokine genes listed in Table 1 was quantified by the 2^−ΔΔCt^ or absolute quantification method. The expression of *ip10*, *il6*, and *il10* in blood; *ip10* in kidney; *il6* in liver; and *il10* in lung tissue were assessed by comparing ΔCt (Ct of target gene − Ct of *rpl18*) between hamsters infected with the serovars Manilae and Hebdomadis because of lack of detectability in control hamsters. All genes, with the exception of *inos* in kidney and liver tissues, were upregulated upon infection with either of the two strains, and maximum expression of most of the genes was detected at 96 h pi (S2, S3, S4, S5 and S6 Figs). The level of expression of *il2*, *il4*, and *ifngamma* was determined by the absolute quantification method. Slight elevation in the expression of *ifngamma* was detected only in the liver tissue of hamsters infected with serovar Manilae at 96 h pi (data not shown). On the other hand, the expression of *il2* and *il4* was below the detection limit in all tissues at any time point irrespective of infection.

Histopathological changes were observed in infected hamsters at 96 h pi. Urinary casts were observed in the kidney tissues of both groups, while degenerative to necrotic changes were evident only in hamsters infected with serovar Manilae, which exhibited severe distortion of tubular cell arrangement and disruption of renal tubules (S7A–S7C Fig). Severe lesions were not observed in liver tissues; however, cloudy swelling of hepatocytes was observed in hamsters infected with serovar Manilae (S7D–S7F Fig). Hemorrhage was observed in lung tissues of hamsters infected with serovar Manilae (S7G–S7I Fig).

Leptospires were detectable in kidney and lung tissues only at 96 h pi, and maximum leptospiral burden was observed in blood and liver at 96 h pi (S1 Fig). In addition, maximum expression of the majority of cytokine genes investigated in tissues was observed at 96 h pi (S2, S3, S4, S5 and S6 Figs). Therefore, leptospiral burden and the levels of expression of cytokine genes in tissues were compared at 96 h pi using a larger sample size in subsequent experiments.

### Comparison of leptospiral burden in tissues of hamsters infected with strains of two *L*. *interrogans* serovars at 96 h pi

**Fig 1:**
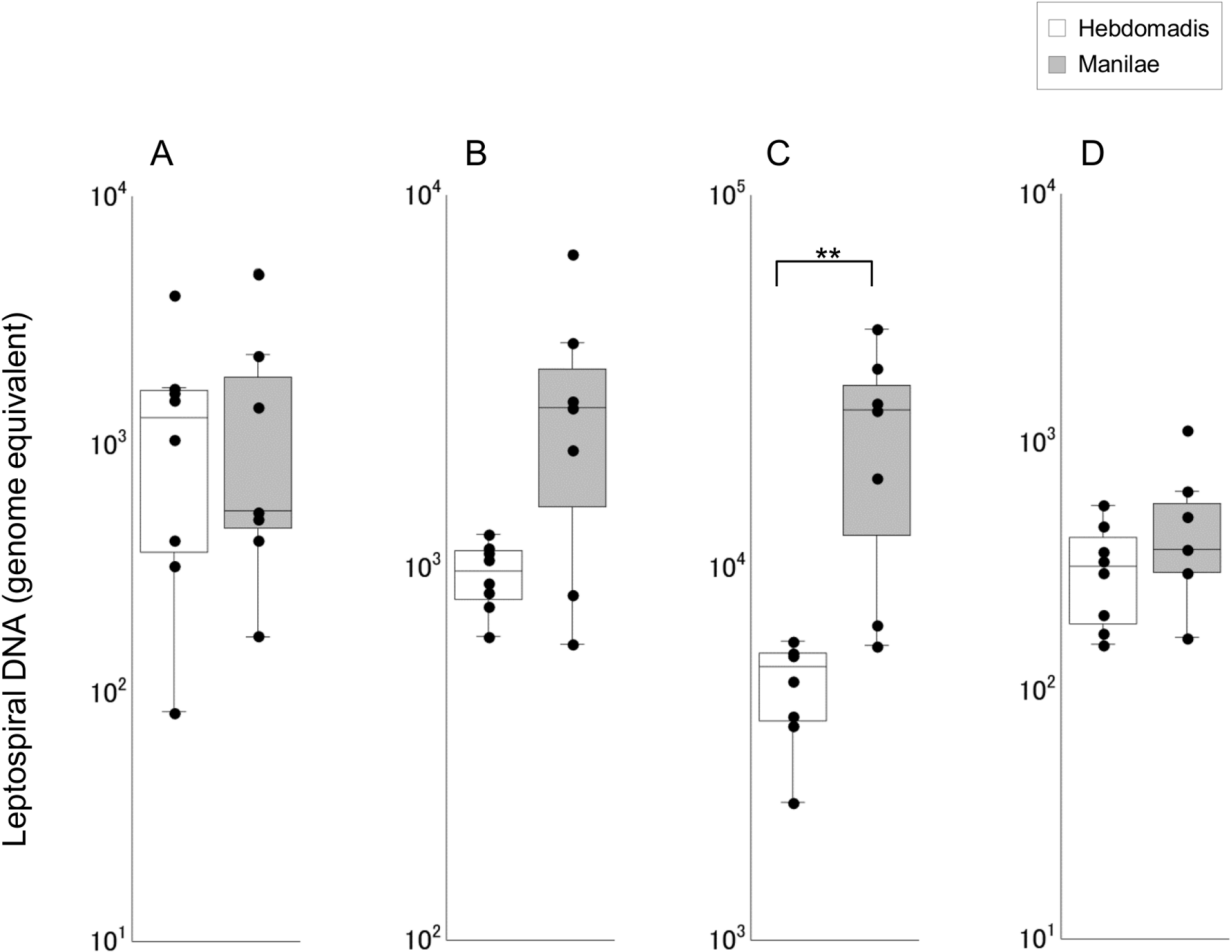
Quantification of leptospiral DNA in tissues of hamsters infected with strains of *L*. *interrogans* serovars Manilae or Hebdomadis at 96 h pi. Leptospiral DNA was quantified by real-time PCR targeting leptospiral *flaB* gene in blood (A), kidney (B), liver (C), and lung (D) tissues of hamsters infected with strains of *L*. *interrogans* serovars Manilae (filled boxes) or Hebdomadis (open boxes) at 96 h pi. The bottom, median, and top lines of the box indicate the 25th, 50th, and 75th percentiles, respectively. The vertical line with whiskers shows the range of values. Experiments were performed in duplicate using two independently extracted DNA samples for each tissue of infected hamsters; each dot indicates the average of two experiments. Data out of the range of values were excluded from statistical analysis. **, p < 0.01.

Leptospiral burden was significantly higher in liver tissues of hamsters infected with serovar Manilae as opposed to serovar Hebdomadis (p < 0.01; Fig 1); significant differences were not observed in other tissues of hamsters infected with strains of the two serovars.

### Comparison of expression levels of cytokine genes in tissues of hamsters infected with strains of two *L*. *interrogans* serovars at 96 h pi

**Fig 2:**
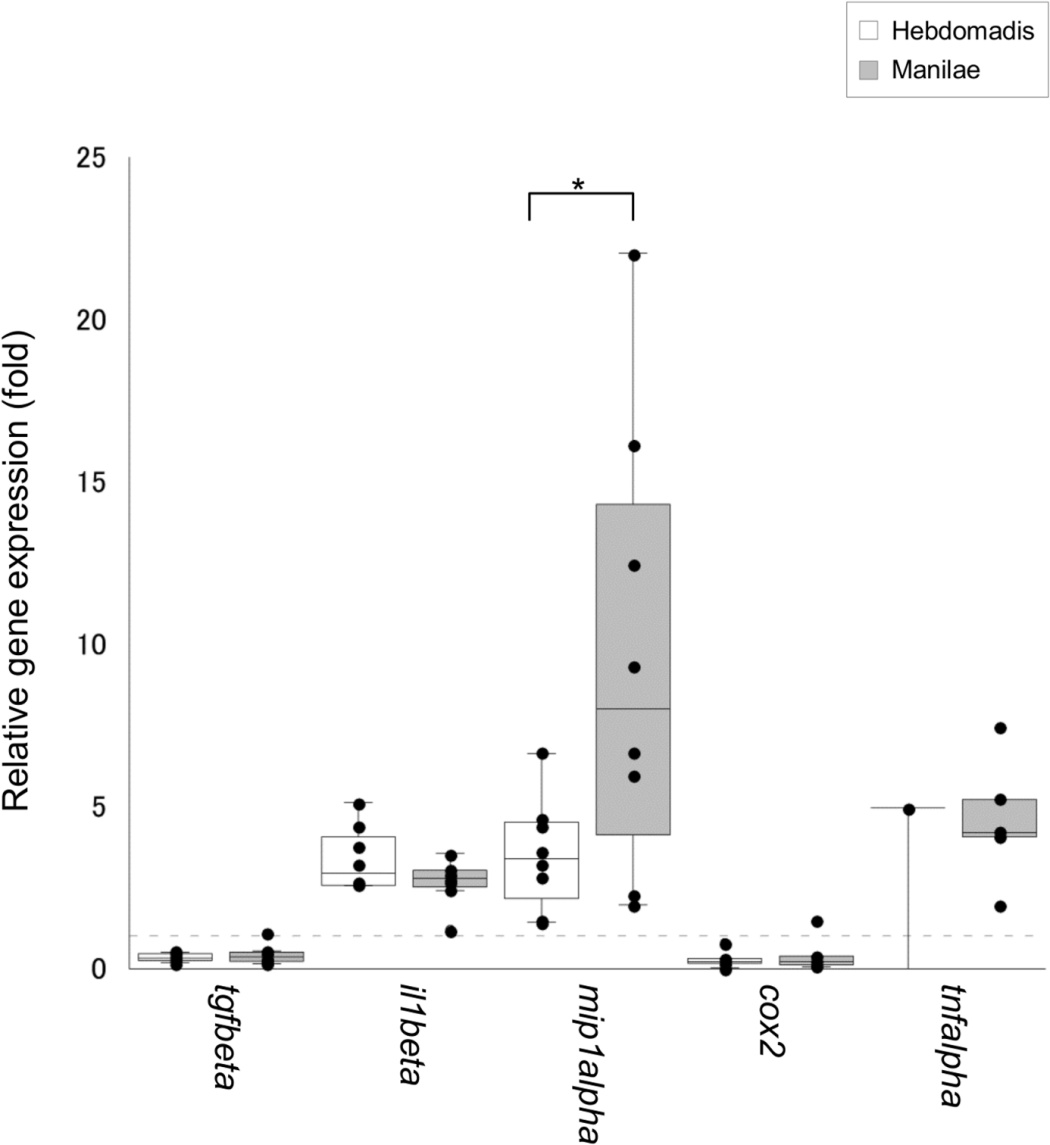
Quantification of expression of cytokine genes in the blood of hamsters infected with *L*. *interrogans* serovars Manilae or Hebdomadis at 96 h pi. Cytokine gene expression in the blood of hamsters infected with strains of *L*. *interrogans* serovars Manilae (filled boxes) or Hebdomadis (open boxes) was quantified at 96 h pi using real-time PCR (2^−ΔΔCt^ method). The bottom, median, and top lines of the box indicate the 25th, 50th, and 75th percentiles, respectively. The vertical line with whiskers shows the range of values. Experiments were performed in duplicate using two independently extracted RNA samples for each hamster; each dot indicates the average of two experiments and gene expression relative to control hamsters. The dotted line indicates the expression level in control hamsters (for calibration). Data out of the range of values were excluded from statistical analysis. *, p < 0.05.

**Fig 3:**
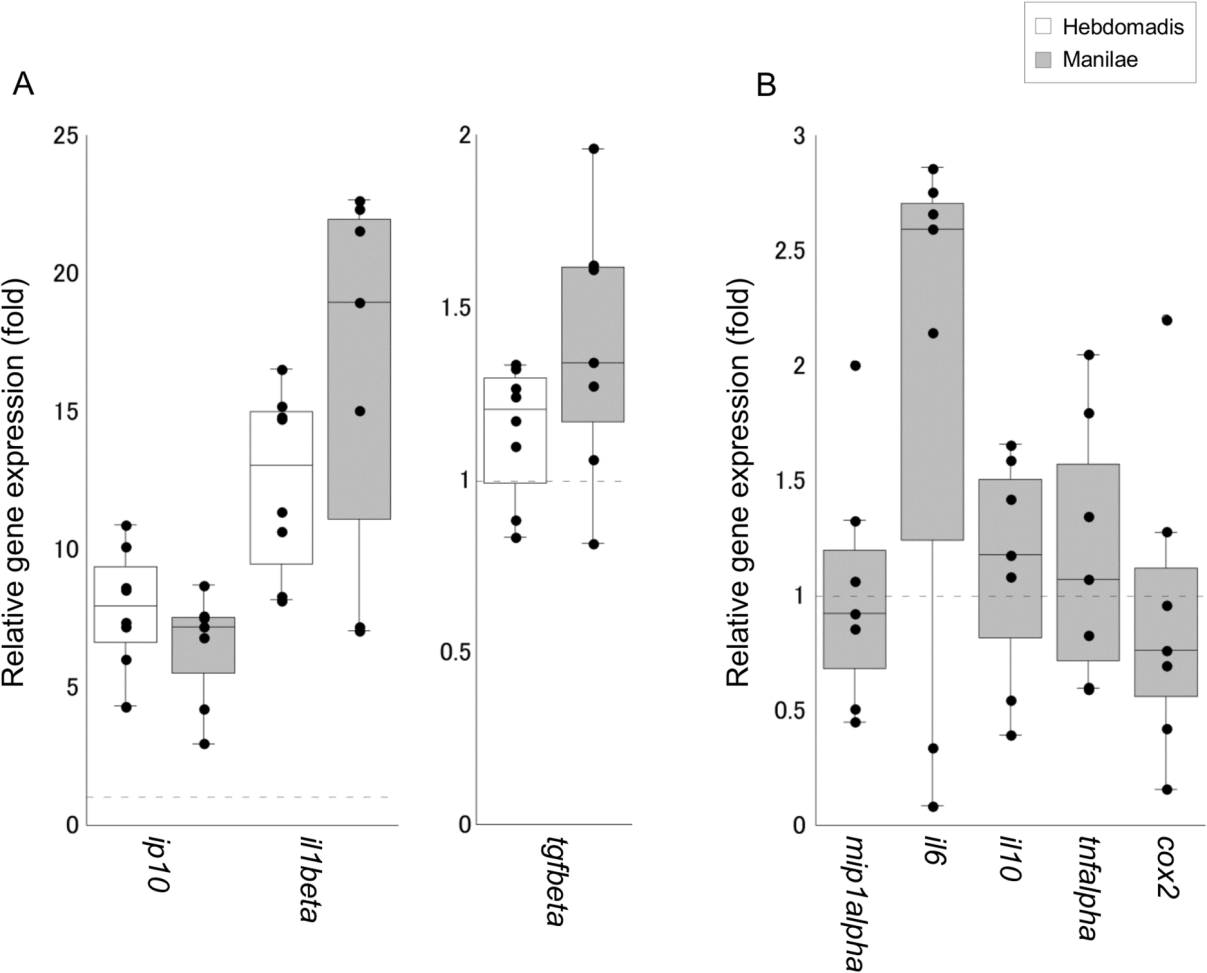
Quantification of cytokine gene expression in kidney tissues of hamsters infected with *L*. *interrogans* serovars Manilae or Hebdomadis at 96 h pi. Cytokine gene expression in kidney tissues of hamsters infected with strains of *L*. *interrogans* serovars Manilae (filled boxes) or Hebdomadis (open boxes) was quantified at 96 h pi using real-time PCR (2^−ΔΔCt^ method). The bottom, median, and top lines of the box indicate the 25th, 50th, and 75th percentiles, respectively. The vertical line with whiskers shows the range of values. The expression of cytokine genes in panel B was below the detection limit in control hamsters. Experiments were performed in duplicate using two independently extracted RNA samples for each hamster; each circle indicates the average of two experiments and gene expression relative to control hamsters (A) or hamsters infected with serovar Hebdomadis (B). The dotted line indicates the expression level in control (A) or Hebdomadis-infected (B) hamsters. Data out of the range of values were excluded from statistical analysis.

**Fig 4:**
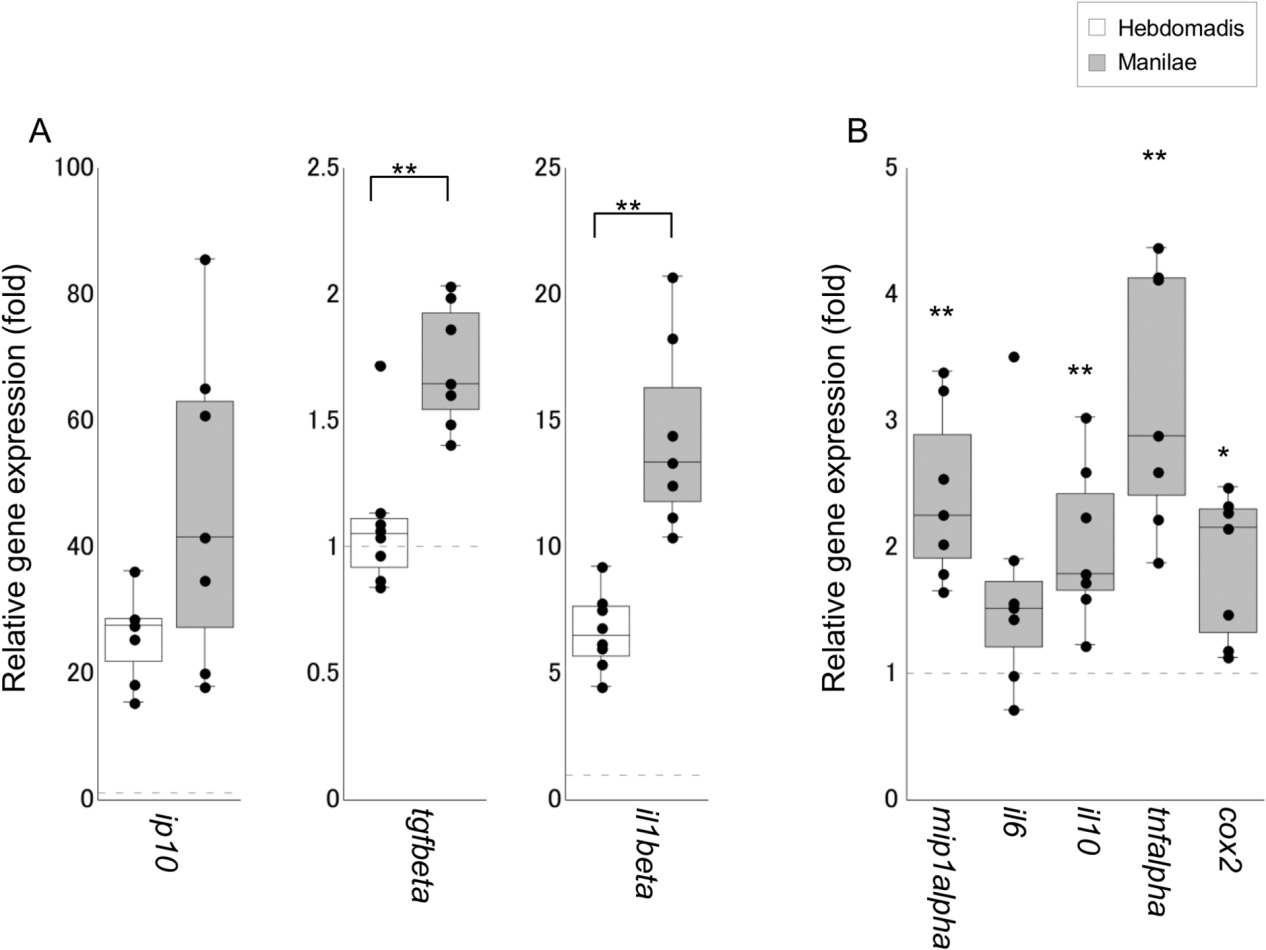
Quantification of cytokine gene expression in liver tissues of hamsters infected with *L*. *interrogans* serovars Manilae or Hebdomadis at 96 h pi. Cytokine gene expression in liver tissues of hamsters infected with strains of *L*. *interrogans* serovars Manilae (filled boxes) or Hebdomadis (open boxes) was quantified at 96 h pi using real-time PCR (2^−ΔΔCt^ method). The bottom, median, and top lines of the box indicate the 25th, 50th, and 75th percentiles, respectively. The vertical line with whiskers shows the range of values. The expression of cytokine genes in panel B was below the detection limit in control hamsters. Experiments were performed in duplicate using two independently extracted RNA samples for each hamster; each circle in the plots indicates the average of two experiments and gene expression relative to control hamsters (A) or hamsters infected with serovar Hebdomadis (B). The dotted line indicates the expression level in control (A) or Hebdomadis-infected (B) hamsters. Data out of the range of values were excluded from statistical analysis. *, p < 0.05; **, p < 0.01; in panel B, the asterisks indicate significant differences in ΔCt values (Ct value of target gene − Ct value of *rpl18*) between hamsters infected with serovars Manilae and Hebdomadis.

**Fig 5:**
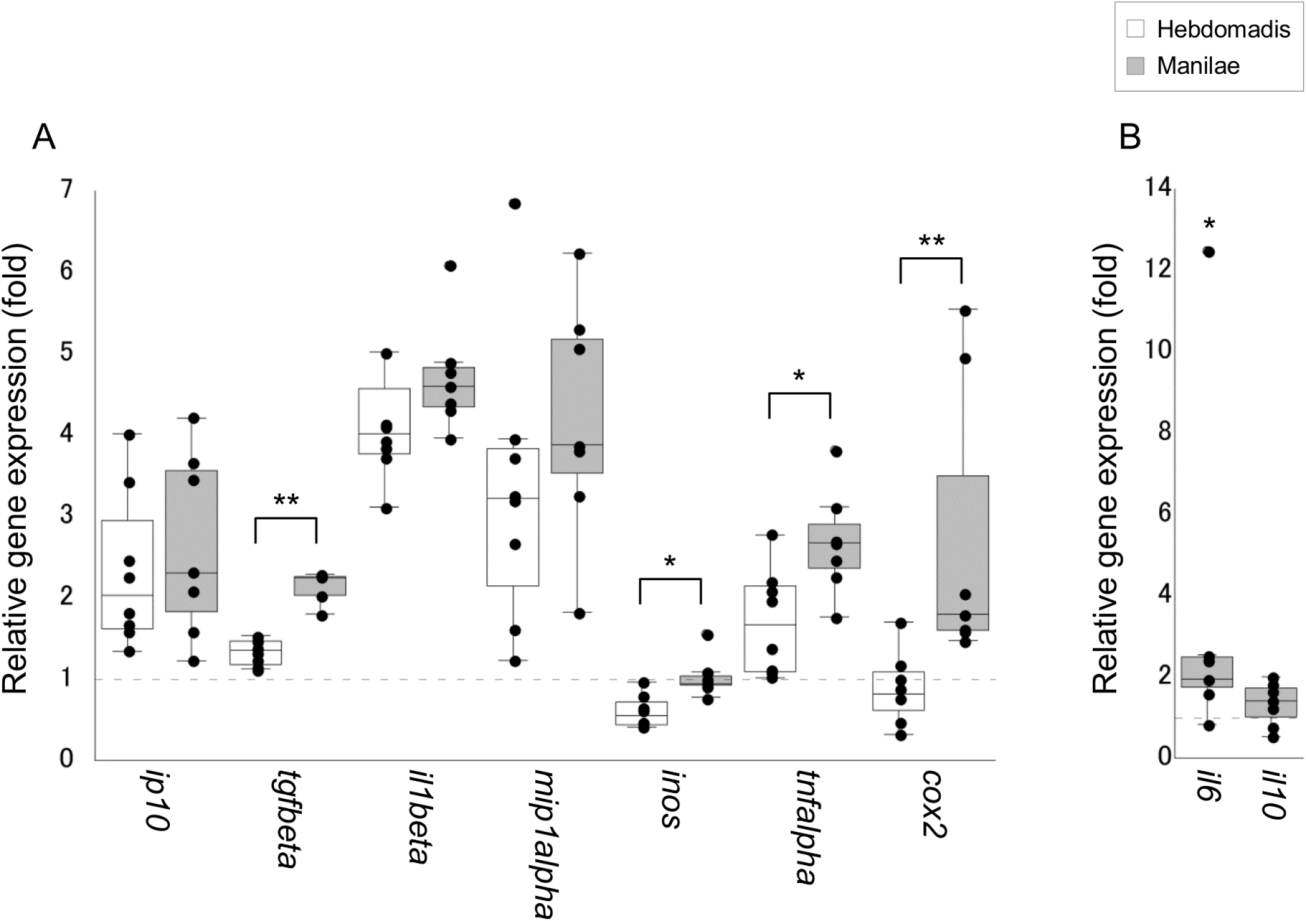
Quantification of cytokine gene expression in lung tissues of hamsters infected with *L*. *interrogans* serovars Manilae or Hebdomadis at 96 h pi. Cytokine gene expression in lung tissues of hamsters infected with strains of *L*. *interrogans* serovars Manilae (filled boxes) or Hebdomadis (open boxes) was quantified at 96 h pi using real-time PCR (2^−ΔΔCt^ method). The bottom, median, and top lines of the box indicate the 25th, 50th, and 75th percentiles, respectively. The vertical line with whiskers shows the range of values. The expression of cytokine genes in panel B was below the detection limit in control hamsters. Experiments were performed in duplicate using two independently extracted RNA samples for each hamster; each circle in the plots indicates the average of two experiments and gene expression relative to control hamsters (A) or hamsters infected with serovar Hebdomadis (B). Dotted line indicates the expression level in control (A) or Hebdomadis-infected (B) hamsters. Data out of the range of values were excluded from statistical analysis. *, p < 0.05; **, p < 0.01; in panel B, the asterisks indicate significant differences in ΔCt values (Ct value of target gene − Ct value of *rpl18*) between hamsters infected with the serovars Manilae and Hebdomadis.

Of the 12 cytokine genes tested, the expression of three genes *il1beta*, *mip1alpha*, and *tgfbeta* was upregulated in the blood of infected hamsters (Fig 2). Moreover, the expression of *mip1alpha* was significantly higher in hamsters infected with serovar Manilae as opposed to serovar Hebdomadis (p < 0.05; Fig 2). In addition, the number of hamsters with detectable expression of *tnfalpha* was significantly higher in the group infected with serovar Manilae (5/7 animals) as opposed to serovar Hebdomadis (1/8 animals; p = 0.04, as determined by Fischer’s Exact test). The expression of eight of the 12 genes tested was upregulated in kidney tissues upon leptospiral infection, although significant differences were not detected between the two serovars Manilae and Hebdomadis (Fig 3). The expression of eight of the 12 genes tested was enhanced in both liver and lung tissues upon leptospiral infection (Figs 4 and 5); significantly higher expression levels of *tgfbeta*, *il1beta*, *mip1alpha*, *il10*, *tnfalpha*, and *cox2* in liver (*cox2*, p < 0.05; other genes, p < 0.01; Fig 4) and of *tgfbeta*, *il6*, *tnfalpha*, and *cox2* in lung (*il6* and *tnfalpha*, p < 0.05; *tgfbeta* and *cox2*, p < 0.01; Fig 5) tissues were observed in the group infected with serovar Manilae as opposed to serovar Hebdomadis. The expression levels of *il2*, *il4*, and *ifngamma* were below the detection limit in all tissues in this round of infection experiments.

## Discussion

The spectrum of clinical symptoms associated with human leptospirosis is extremely broad, ranging from asymptomatic or mild influenza-like illness to severe fatal disease [2, 4]. The broad range of disease severity has been attributed to differences in virulence determinants between various serotypes or genotypes of *Leptospira* spp. [17–19]. Apart from acute incidental infection, each *Leptospira* serovar is usually associated with a particular maintenance host in which an associated serovar causes asymptomatic or only mild manifestation [2]. Several possible determinants of leptospirosis severity have been suggested though [9], the mechanism underlying the difference in virulence remains to be deciphered. In the present study, bacterial burden and cytokine expression profiles were compared between hamsters infected with strains belonging to the *L*. *interrogans* serovars Manilae and Hebdomadis.

The leptospiral burden in liver tissues was significantly higher in hamsters infected with serovar Manilae than those infected with serovar Hebdomadis (Fig 1). Two hundred nanograms of DNA extracted from hamster tissues is equivalent to approximately 150 μg of kidney and liver tissues and approximately 500 μg of lung tissues [23]. Thus, leptospiral burden per unit tissue weight was highest for the liver tissue. The average leptospiral genome copy numbers in blood and liver tissues of hamsters infected with serovar Manilae were 1,302 and 20,559, respectively, compared with 1,340 and 4,896, respectively, in hamsters infected with serovar Hebdomadis. Significant differences in leptospiral genome copy numbers were not observed between hamsters infected with serovars Manilae and Hebdomadis in blood, kidney, and lung tissues. These results indicate that *L*. *interrogans* serovar Manilae multiplied with greater efficiency in the liver tissue. The liver-specific macrophages, Kupffer cells (KCs), play a central role in pathogen elimination [24]. Previous studies reported the survival of pathogenic leptospires in KCs and macrophages from humans, rats, and guinea pigs [25–27]. *L*. *interrogans* serovar Manilae strain was also capable of multiplying and escaping the action of mouse macrophages and was eliminated with lower efficiency *ex vivo* than a nonpathogenic strain of *L*. *biflexa* [28]. Therefore, *L*. *interrogans* serovar Manilae strain is presumed to possess the ability to escape the bactericidal action of KCs and multiply more efficiently in liver tissues than serovar Hebdomadis.

Previous studies demonstrated elevation in the levels of both pro- and anti-inflammatory cytokines in leptospirosis, which is more prominent in patients with severe disease [10–15]. Several cytokines, such as IL-6, IL-10, and TNF-α, have been shown to be associated with the severity of the disease [11–13]. In addition, experimental infection revealed significant elevation in the expression of *tnfalpha*, *il1alpha*, *cox2*, and *il10* in the blood of dead hamsters compared with that of the survivors [16]. The present study also revealed upregulation of genes encoding both pro- and anti-inflammatory cytokines in infected hamsters (Figs 2, 3, 4 and 5), which is similar to the scenario in human patients with severe leptospirosis as well as hamster models of leptospirosis. In addition, the expression levels of inflammatory cytokines, such as *il6* in lungs, *il10* in liver; *tnfalpha* in blood, liver, and lung tissues; and *cox2* in liver and lungs, all of which are considered to be relevant to the severity of leptospirosis, were significantly higher in hamsters infected with serovar Manilae compared with serovar Hebdomadis (Figs 2, 3, 4 and 5; see Results section). These results suggest that both serovars of *L*. *interrogans* elicit systemic inflammatory response in hamsters, and the stronger induction of genes encoding inflammatory cytokines upon infection with serovar Manilae is responsible for the difference in virulence of these two serovars.

Several leptospiral factors are known to induce the production of cytokines or the expression of cytokine genes [29–34]. Leptospiral lipopolysaccharide (LPS) activates monocytes through Toll-like receptor 2, releasing inflammatory cytokines [29]. A recent report showed that *L*. *interrogans* serovar Lai altered the structure of lipid A, a constituent of LPS responsible for its toxicity, in human and murine macrophages; this resulted in differential cytokine gene expression [30]. Leptospiral lipoprotein LipL32 and hemolysins have been shown to induce *inos*, *tnfalpha*, and *ccl2* expression in cultured mouse renal proximal tubule cells and IL-6, IL-1β, and TNF-α production in cultured human and murine macrophages [31–33]. Injection of leptospiral glycoprotein GLP into mouse lung has been shown to trigger the production of IL-6, IL-1β, TNF-α, and MIP-1α [34]. The genome copy number of serovar Manilae was not significantly different from that of serovar Hebdomadis in blood, kidney, and lung. Therefore, differences in cytokine gene expression profile between hamsters infected with the serovars Manilae and Hebdomadis are likely to contribute to structural and/or quantitative differences in these molecules between the strains. In addition, the strain belonging to serovar Manilae could possess unique molecules that synergistically induce cytokine gene expression.

Histopathological changes were also observed in infected hamsters. Severe destruction of renal tubule cells was observed in the kidneys of hamsters infected with serovar Manilae (S7A–S7C Fig); however, significant differences were not observed in bacterial burden and cytokine gene expression profiles between hamsters infected with serovars Manilae and Hebdomadis (Fig 1). Hemorrhage was observed in the lungs of hamsters infected with serovar Manilae (S7G–S7I Fig); this group also exhibited significantly higher expression of certain cytokine genes than hamsters infected with serovar Hebdomadis (Fig 5). The experimental infection of hamsters with strains of *L*. *interrogans* serovar Icterohaemorrhagiae also resulted in pulmonary hemorrhage and elevated expression of *tnfalpha* and *enos* [6]. In addition, the level of serum TNF-α was higher in leptospirosis patients with pulmonary hemorrhage, and serum IL-6 level was associated with fatalities from severe pulmonary hemorrhage syndrome [11, 12]. However, in a mouse model of pulmonary hemorrhage, the expression of *il1beta*, *tnfalpha*, and *tgfbeta* was found to increase subsequent to hemorrhage [35]. Whether the upregulated expression of cytokine genes or increased cytokine production observed in the present as well as previous studies on human and experimental leptospirosis is a mediator or marker of pulmonary hemorrhage remains unclear. Moreover, bacterial burden in lung tissue was not significantly different between hamsters infected with serovars Manilae and Hebdomadis (Fig 1). These results suggest a serovar Manilae-specific virulence mechanism that provokes renal damage and pulmonary hemorrhage.

Although bacterial burden and expression of certain cytokine genes were significantly higher in the liver tissues of hamsters infected with serovar Manilae than serovar Hebdomadis (Figs 1 and 4), significant difference in lesions was not observed in the liver (S7D–S7F Fig). Therefore, damage to liver tissues is probably not attributable to the difference in pathogenesis between serovars Manilae and Hebdomadis.

There are some limitations in this study. The study compared the expression of the limited number of cytokine genes (12 genes) at a single time point and single infectious dose. Comparison of different challenge doses and different (later) time points as well as a broader range of cytokine genes would give more insight into immune response against leptospiral infection and immune evasion mechanisms by *L*. *interrogans*. There is a possibility that our data include animals that may have survive if not sacrificed. However, our previous studies demonstrate that 10^6^ cells of the serovar Manilae strain is almost always fatal to hamsters but the serovar Hebdomadis is not.

In summary, the strain belonging to serovar Manilae multiplied with greater efficiency in liver tissues and induced higher expression of genes encoding both pro- and anti-inflammatory cytokines than serovar Hebdomadis even in tissues in which a significant difference in leptospiral load was not observed. The results obtained in this study suggest that the strain belonging to serovar Manilae possesses a unique virulence mechanism responsible for inducing severe damage to kidney and lung tissues. Elucidation of the underlying molecular mechanism is indispensable for understanding the pathogenesis of severe leptospirosis.

## Supporting Information

S1 ARRIVE Guidelines Checklist (PDF)

S1 Fig Temporal changes in leptospiral DNA in tissues of hamsters infected with *L*. *interrogans* serovars Manilae or Hebdomadis. Leptospiral DNA was quantified by real-time PCR targeting *flaB* in blood (A), kidney (B), liver (C), and lung (D) tissues of hamsters infected with strains of *L*. *interrogans* serovars Manilae (filled circles) or Hebdomadis (open circles) at 12, 24, 48, 72, and 96 h pi. Experiments were performed in duplicate using two independently extracted DNA samples for each tissue of infected hamsters. Each circle indicates the average of two experiments.
(PDF)

S2 Fig Temporal changes in cytokine gene expression in the blood of hamsters infected with *L*. *interrogans* serovars Manilae or Hebdomadis. Cytokine gene expression in the blood of hamsters infected with strains of *L*. *interrogans* serovars Manilae (filled circles) or Hebdomadis (open circles) was quantified at 12, 24, 48, 72, and 96 h pi using real-time PCR (2^−ΔΔCt^ method). Experiments were performed in duplicate using two independently extracted RNA samples for each hamster; each circle indicates the average of two experiments and gene expression relative to control hamsters. The dotted line indicates the expression level in control hamsters (for calibration).
(PDF)

S3 Fig Temporal changes in cytokine gene expression in kidney tissues of hamsters infected with *L*. *interrogans* serovars Manilae or Hebdomadis. Cytokine gene expression in kidney tissues of hamsters infected with strains of *L*. *interrogans* serovars Manilae (filled circles) or Hebdomadis (open circles) was quantified at 12, 24, 48, 72, and 96 h pi using real-time PCR (2^−ΔΔCt^ method). Experiments were performed in duplicate using two independently extracted RNA samples for each hamster; each circle indicates the average of two experiments and gene expression relative to control hamsters. The dotted line indicates the expression level in control hamsters (for calibration).
(PDF)

S4 Fig Temporal changes in cytokine gene expression in liver tissues of hamsters infected with *L*. *interrogans* serovars Manilae or Hebdomadis. Cytokine gene expression in liver tissues of hamsters infected with strains of *L*. *interrogans* serovars Manilae (filled circles) or Hebdomadis (open circles) was quantified at 12, 24, 48, 72, and 96 h pi using real-time PCR (2^−ΔΔCt^ method). Experiments were performed in duplicate using two independently extracted RNA samples for each hamster; each circle indicates the average of two experiments and gene expression relative to control hamsters. The dotted line indicates the expression level in control hamsters (for calibration).
(PDF)

S5 Fig Temporal changes in cytokine gene expression in lung tissues of hamsters infected with *L*. *interrogans* serovars Manilae or Hebdomadis. Cytokine gene expression in lung tissues of hamsters infected with strains of *L*. *interrogans* serovars Manilae (filled circles) or Hebdomadis (open circles) was quantified at 12, 24, 48, 72, and 96 h pi using real-time PCR (2^–ΔΔCt^ method). Experiments were performed in duplicate using two independently extracted RNA samples for each hamster. Each circle indicates the average of two experiments and gene expression relative to control hamsters. The dotted line indicates the expression level in control hamsters (for calibration).
(PDF)

S6 Fig Temporal changes in cytokine gene expression in tissues of hamsters infected with strains of *L*. *interrogans* serovars Manilae or Hebdomadis. Expression levels of cytokine genes in tissues of hamsters infected with *L*. *interrogans* serovars Manilae (filled circles) or Hebdomadis (open circles) were expressed as ΔCt (Ct value of target gene − Ct value of *rpl18*). (A) ΔCt of *ip-10*, *il-6*, and *il-10* in blood at 72 and 96 h pi; (B) ΔCt of *ip-10* in kidney tissues at 96 h pi; (C) ΔCt of *il-6* in liver tissues at 96 h pi; and (D) ΔCt of *il-10* in lung tissues at 72 and 96 h pi. Experiments were performed in duplicate using two independently extracted RNA samples for each hamster. Each circle indicates the average of two experiments.
(PDF)

S7 Fig Histological lesions in hamsters infected with L. interrogans serovars Manilae or Hebdomadis at 96 h pi. Hematoxylin-and-eosin-stained sections of kidney (A–C), liver (D–F), and lung (G–I) tissues from naïve (A, D and G), serovar Hebdomadis-infected (B, E and H) and serovar Manilae-infected (C, F and I) hamsters were microscopically observed. Arrows indicate urinary casts (B, C) and arterial blood (G–I). Asterisks indicate central vein (D–F) and edema (G–I). The dotted circle (I) indicates hemorrhage in lung tissue. Magnification: 200x (A–F), 100x (G–I).
(PDF)
