# Supplementary material for: Comparison of Bacterial Burden and Cytokine Gene Expression in Golden Hamsters in Early Phase of Infection with Two Different Strains of Leptospira interrogans
Source: PLoS One. 2015 Jul 6;10(7):e0132694. doi: 10.1371/journal.pone.0132694 (PMC4492770; doi:10.1371/journal.pone.0132694)
Supplement: S1 Fig — Leptospiral DNA was quantified by real-time PCR targeting flaB in blood (A), kidney (B), liver (C), and lung (D) tissues of hamsters infected with strains of L. interrogans serovars Manilae (filled circles) or Hebdomadis (open circles) at 12, 24, 48, 72, and 96 h pi. Experiments were performed in duplicate using two independently extracted DNA samples for each tissue of infected hamsters. Each circle indicates the average of two experiments. (PDF) [file pone.0132694.s002.pdf]

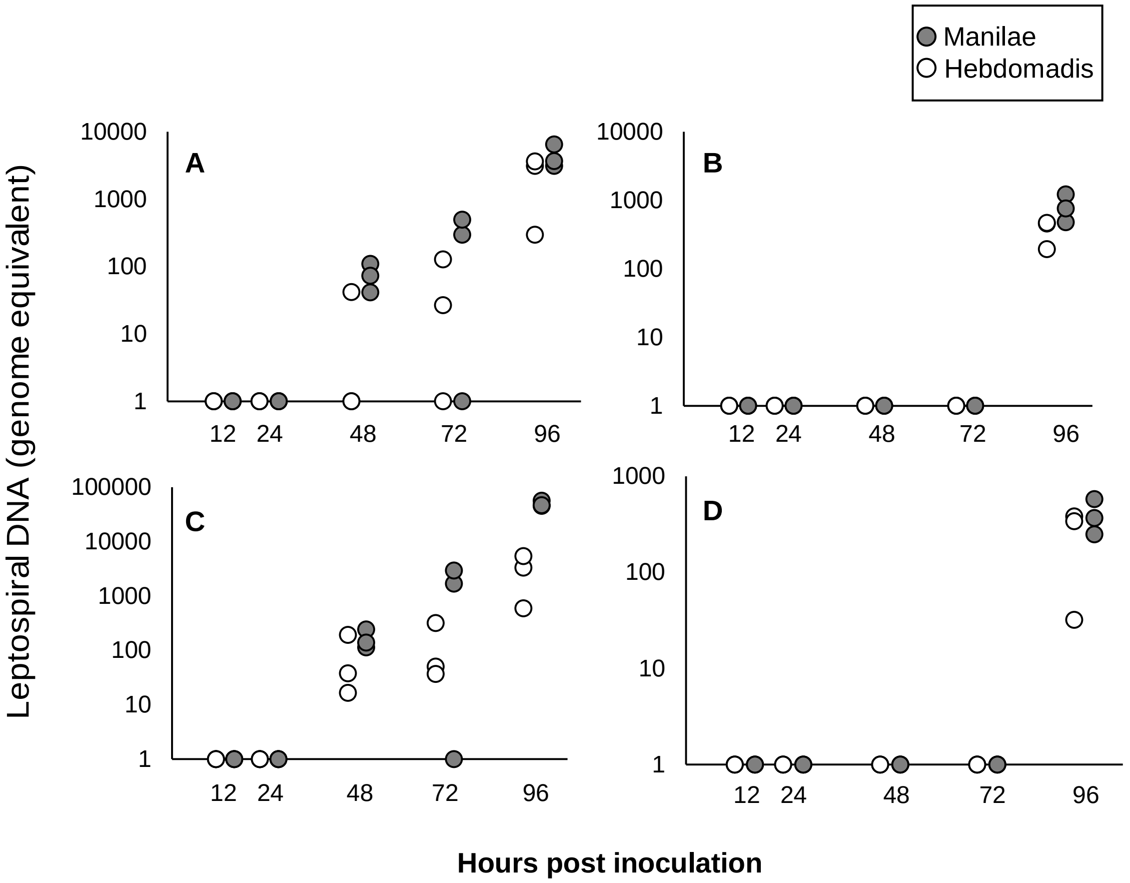

S1 Fig. Temporal change of leptospiral DNA in tissues of hamsters infected with serovars Manilae or Hebdomadis strains.
