# Supplementary material for: Comparison of Bacterial Burden and Cytokine Gene Expression in Golden Hamsters in Early Phase of Infection with Two Different Strains of Leptospira interrogans
Source: PLoS One. 2015 Jul 6;10(7):e0132694. doi: 10.1371/journal.pone.0132694 (PMC4492770; doi:10.1371/journal.pone.0132694)
Supplement: S5 Fig — Cytokine gene expression in lung tissues of hamsters infected with strains of L. interrogans serovars Manilae (filled circles) or Hebdomadis (open circles) was quantified at 12, 24, 48, 72, and 96 h pi using real-time PCR (2–ΔΔCt method). Experiments were performed in duplicate using two independently extracted RNA samples for each hamster. Each circle indicates the average of two experiments and gene expression relative to control hamsters. The dotted line indicates the expression level in control hamsters (for calibration). (PDF) [file pone.0132694.s006.pdf]

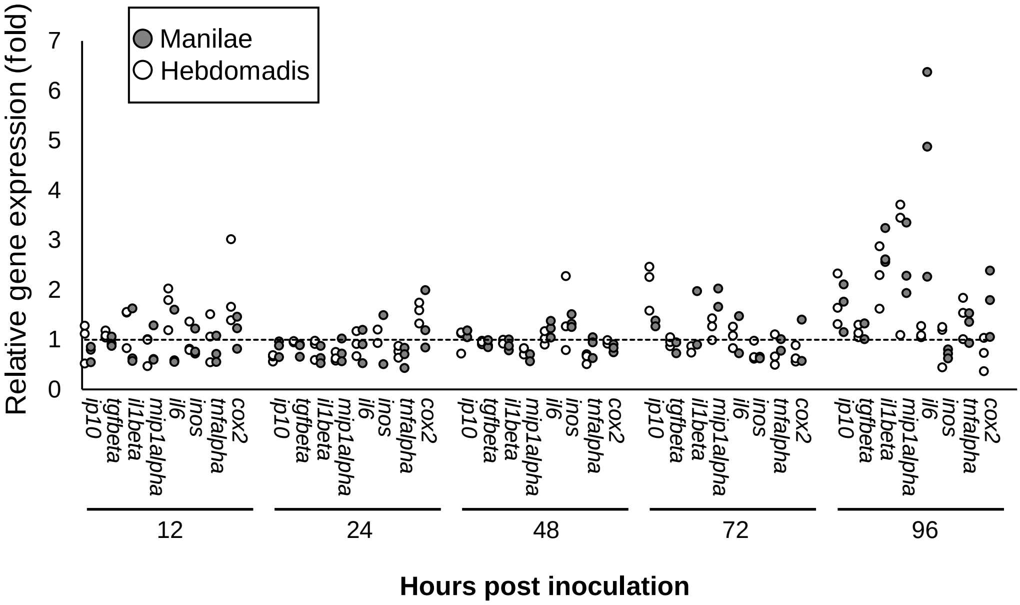

S5 Fig. Temporal change of cytokine gene expressions in lung of hamsters infected with serovars Manilae or Hebdomadis strains.
