# Supplementary material for: Comparison of Bacterial Burden and Cytokine Gene Expression in Golden Hamsters in Early Phase of Infection with Two Different Strains of Leptospira interrogans
Source: PLoS One. 2015 Jul 6;10(7):e0132694. doi: 10.1371/journal.pone.0132694 (PMC4492770; doi:10.1371/journal.pone.0132694)
Supplement: S6 Fig — Expression levels of cytokine genes in tissues of hamsters infected with L. interrogans serovars Manilae (filled circles) or Hebdomadis (open circles) were expressed as ΔCt (Ct value of target gene − Ct value of rpl18). (A) ΔCt of ip-10, il-6, and il-10 in blood at 72 and 96 h pi; (B) ΔCt of ip-10 in kidney tissues at 96 h pi; (C) ΔCt of il-6 in liver tissues at 96 h pi; and (D) ΔCt of il-10 in lung tissues at 72 and 96 h pi. Experiments were performed in duplicate using two independently extracted RNA samples for each hamster. Each circle indicates the average of two experiments. (PDF) [file pone.0132694.s007.pdf]

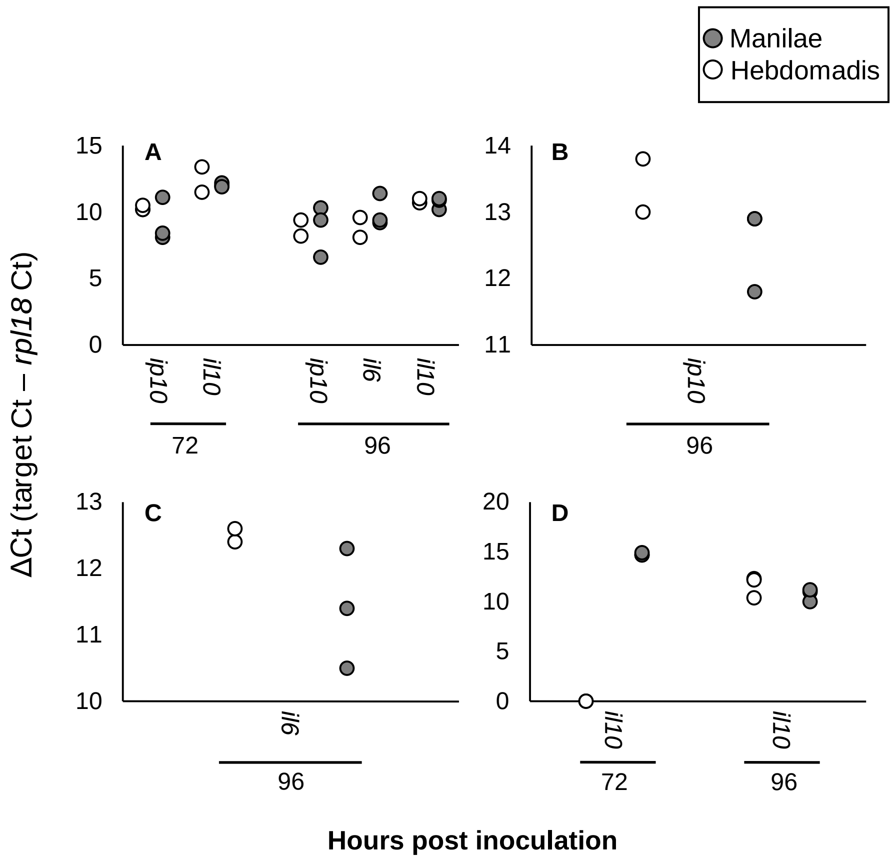

S6 Fig. Temporal change of cytokine gene expressions in tissues of hamsters infected with serovars Manilae or Hebdomadis strains.
