# Supplementary material for: Comparison of Bacterial Burden and Cytokine Gene Expression in Golden Hamsters in Early Phase of Infection with Two Different Strains of Leptospira interrogans
Source: PLoS One. 2015 Jul 6;10(7):e0132694. doi: 10.1371/journal.pone.0132694 (PMC4492770; doi:10.1371/journal.pone.0132694)
Supplement: S7 Fig — Hematoxylin-and-eosin-stained sections of kidney (A–C), liver (D–F), and lung (G–I) tissues from naïve (A, D and G), serovar Hebdomadis-infected (B, E and H) and serovar Manilae-infected (C, F and I) hamsters were microscopically observed. Arrows indicate urinary casts (B, C) and arterial blood (G–I). Asterisks indicate central vein (D–F) and edema (G–I). The dotted circle (I) indicates hemorrhage in lung tissue. Magnification: 200x (A–F), 100x (G–I). (PDF) [file pone.0132694.s008.pdf]

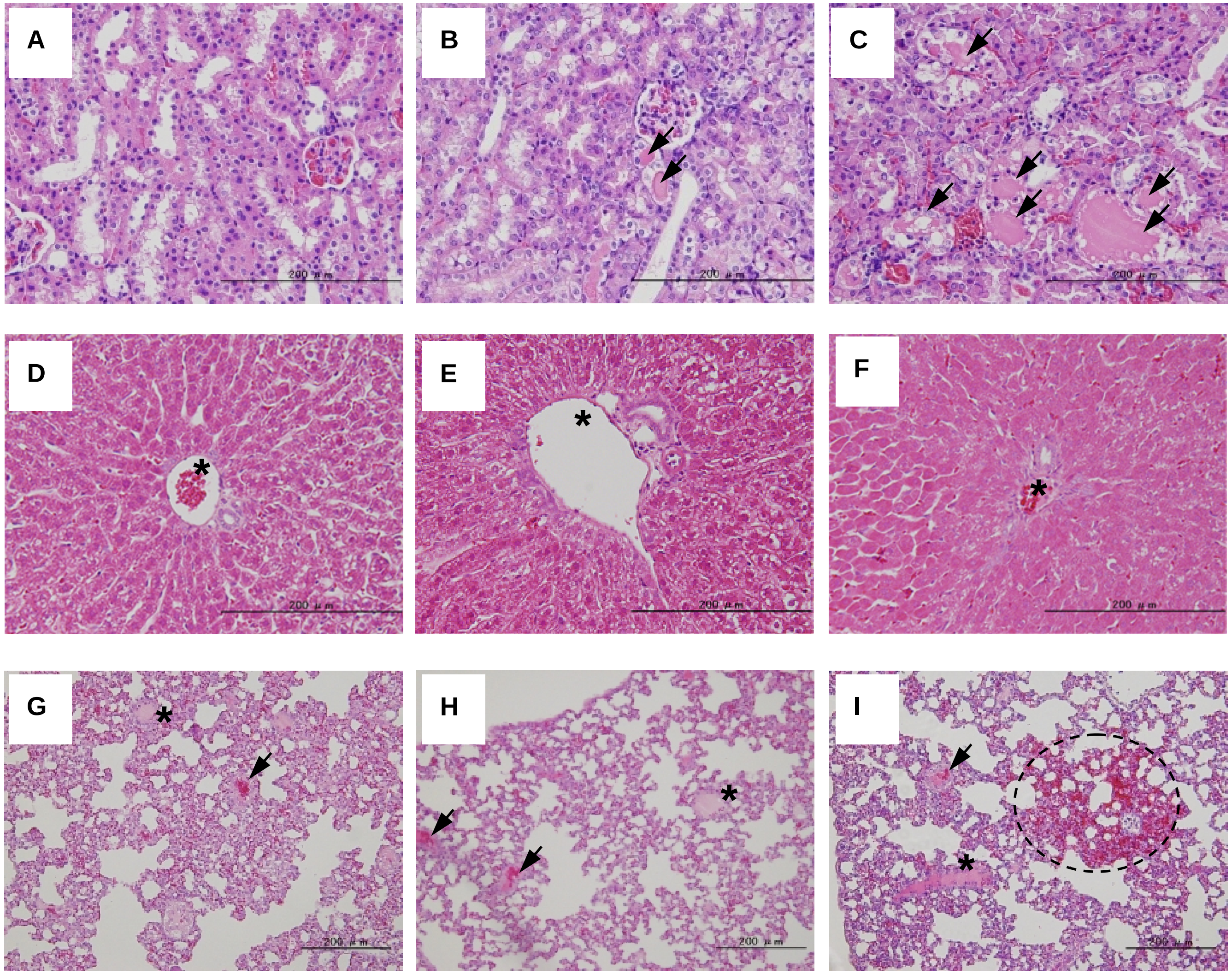

S7 Fig. Histological lesions in hamsters infected with serovar Manilae or Hebdomadis strains at 96 h post inoculation (hematoxylin and eosin stain).
